# Association Between Fear of Progression and Dyadic Self-Care in Patients With Stroke and Their Spouses: A Moderated Network Analysis

**DOI:** 10.1097/jnr.0000000000000770

**Published:** 2026-08-19

**Authors:** Luyi XU, Tingting LIN, Zheng WANG, Siyi SU, Linchao YU, Yuji YUAN, Shishi DONG, Jufang LI, Ping LI

**Affiliations:** 1 School of Nursing, Wenzhou Medical University, Wenzhou, Zhejiang, China; 2 The Second Affiliated Hospital of Wenzhou Medical University

**Keywords:** dyadic self-care, fear of disease progression, stroke, moderated network model.

## Abstract

**Background:**

Self-care, while a vital factor affecting health outcomes in patients with stroke, remains unsatisfactory in this patient population. The dyadic self-care concept offers new research directions. Although fear of disease progression is known to affect self-care significantly, the focus of prior research has been primarily on macro-level variables, and micro-level perspectives have been inadequately explored.

**Purpose:**

This study was designed to explore the relationship between fear of disease progression and dyadic self-care in patients with stroke and their spouses and the moderating role of sex on this relationship.

**Methods:**

From August 2024 to March 2025, 384 stroke patient-spouse dyads completed surveys, including the Fear of Progression Questionnaire Short Form, the Fear of Progression Questionnaire Short Form/Partner, the Self-Care of Chronic Illness Inventory, and the Caregiver Contribution to Self-Care Chronic Illness Inventory. Moderated network analysis was used to explore the bidirectional relationships between fear of disease progression dimensions and dyadic self-care and the moderating role of sex.

**Results:**

The relationships between the patient’s and spouse’s disease progression dimensions and those between the patients’ self-care dimensions and spouses’ contribution to self-care dimensions were shown to be bidirectionally positively correlated. Fear of disease progression dimensions were shown to be bidirectionally negatively correlated with the dyadic self-care dimensions. Fear of disease progression dimensions in male patients were shown to influence their spouses’ fear of disease progression dimensions negatively, while the impact was positive in female patients. Fear of disease progression in female spouses was found to have a stronger negative impact on their contribution to self-care than in male spouses.

**Conclusions/Implications for Practice:**

Fear of disease progression and dyadic self-care are dyadic phenomena in patients with stroke and their spouses. Fear of disease progression is bidirectionally and negatively associated with dyadic self-care, moderated by sex. For male patients, the couple’s emotional regulation is “complementary,” whereas for female patients, this regulation is “covariant.” For male patient–female spouse families, the male patient should be helped to redefine strength and proactively inquire about his spouse’s concerns to provide her with dedicated psychological support to express her emotions. For female patient–male spouse families, open communication should be affirmed while cautioning against excessive emotional contagion, and couple emotion regulation skills should be taught. As excessive fear in female spouses has a stronger negative impact on their contribution to self-care than male spouses, future interventions should assist female spouses to identify and manage invisible emotional labor, to establish “emotional breaks,” and to share emotional support and caregiving tasks with other family members or support systems. Support groups should be created that provide safe spaces for emotional expression and experience sharing. Finally, remote monitoring technologies may be employed to assist patients in improving their self-management efficacy.

## Introduction

Stroke is the second leading cause of death and a major cause of disability worldwide, and the leading cause of death and disability among adults in China (Report on Stroke Center in China Writing Group, 2024). About 75%–80% of patients with stroke face functional impairments in the post-acute phase (GBD 2019 Stroke Collaborators, 2021). Due to the shortage of medical resources, home-based rehabilitation has become the primary form of rehabilitation for patients with stroke (Mahmood et al., 2022). Spouses, the primary caregivers of patients with stroke during home-based rehabilitation, provide emotional support and rehabilitation care and also bear the primary burdens of care and psychological stress (Gurková et al., 2023; Y. Liu et al., 2024).

Self-care refers to the process by which patients maintain health through health-promoting practices and disease management (Riegel et al., 2018). Self-care is crucial to improving the physical and mental health outcomes of patients with stroke and is a core strategy for global health systems to address the growing burden of stroke (Duncan et al., 2021). However, self-care among patients with stroke remains suboptimal (Duncan et al., 2021). The Dyadic Illness Management Theory and related studies that conceptualize self-care behavior as a dyadic phenomenon have proposed the concept of dyadic self-care (B. Liu et al., 2025; Lyons & Lee, 2018). Dyadic self-care involves both patient self-care and caregiver contributions to self-care. Patient self-care behaviors influence the contributions of the caregiver and vice versa. Most patients with stroke rely on their spouses for support after discharge, with spouses being the primary caregivers (Gurková et al., 2023; Y. Liu et al., 2024). Thus, enhancing spousal contributions to the patient's self-care may improve the self-care behaviors in patients, thus improving patient outcomes and reducing recurrence rates.

Fear of disease progression (FoP) refers to fears related to existing illnesses, including concerns about adverse physical, psychological, and social consequences as well as about disease recurrence (Dankert et al., 2003). The findings of prior research confirm that patients with stroke as well as their spouses experience high levels of fear of disease progression, and that a positive correlation exists between the fear of disease progression levels in the two groups (Zhang et al., 2024). This suggests FoP may be a dyadic phenomenon, with the fear level of one influencing that in the other.

FoP in patients and their spouses may interact bidirectionally with dyadic self-care. Previous research findings have confirmed that fear of disease progression can affect patient self-care efficacy and, ultimately, impact their quality of life (Hu & Xu, 2024). In the context of patients with cancer, patient spouses report that caregiving stress during involvement in patient self-care can hinder their ability to assist (Tauber et al., 2019). Thus, spouses’ FoP, a common stressor, likely impedes the effectiveness of the support they provide to patients, which reduces their self-care contributions and ultimately influences patients’ self-care levels. In addition, enhancing self-management skills in patients with cancer has proven to reduce their fear of disease (L. P. Wang et al., 2024). This conclusion must be further verified in patients with stroke and their spouses.

Sex may interact with FoP in patients and their spouses to influence dyadic self-care, and it may also interact with dyadic self-care to influence FoP in patients and their spouses. Female patients are more prone to maladaptive fear and hypochondriacal anxiety in response to stress, resulting in higher levels of FoP (Cui, 2022). Previous research findings indicate female spouses, who often take on greater emotional and caregiving roles, experience heavier psychological burdens and are more sensitive to the health of their care recipient, leading to higher fear levels (Qin et al., 2024). Excessive FoP in female patients or their spouses can foster overwhelming anxiety that impairs their ability to focus on self-care or contribute to the self-care of their stroke-affected spouse. Male patients’ self-care behaviors are not as high as those of female patients (B. B. Johnson et al., 2025), which may lead male patients to take fewer preventive measures, thus increasing disease progression risk and exacerbating FoP in both the patient and their spouse. In traditional family models, women typically assume more responsibility for housework and caregiving, and female spouses contribute more to self-care than male spouses (B. Wang et al., 2024). Thus, female caregivers may provide more self-care support to male patients, potentially alleviating disease progression and reducing FoP in both.

**Figure 1:**
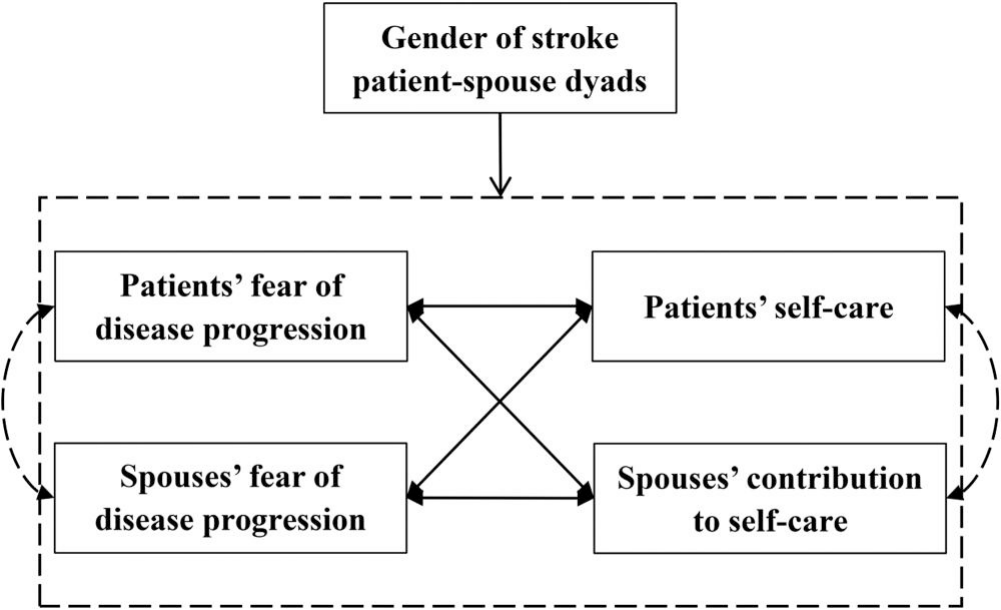
Schematic Diagram of Research Hypotheses

In summary, elucidating the mechanisms of FoP and dyadic self-care in patients with stroke and their spouses, as well as the moderating role of sex, can guide the use of dyadic interventions to improve self-care in patients with stroke and reduce recurrence. However, prior related research has often focused exclusively on either patients or their spouses, and primarily examined overall variables. The moderated network model (MNM) represents a more scientific approach to studying the role of moderator variables within a network from a microscopic perspective (Swanson, 2020). Therefore, in this study, an MNM was constructed to examine the relationships among these variables and the moderating role of sex. The conceptual framework of this study is illustrated in Figure 1, and the research hypotheses are as follows:

- Hypothesis 1: FoP in patients with stroke and their spouses, as well as patients’ self-care and spouses’ contribution to self-care, interact with and influence each other (as indicated by the black dashed line in Figure 1).
- Hypothesis 2: FoP in patients with stroke and their spouses is bidirectionally associated with dyadic self-care (as shown by the black solid line in Figure 1).
- Hypothesis 3: The relationship between FoP and dyadic self-care in patients with stroke and their spouses is moderated by sex.

## Methods

### Design and Participants

In this cross-sectional study, purposive sampling was employed to select stroke patient-spouse caregiver dyads from one tertiary hospital. Data from 384 stroke patient-spouse caregiver dyads were collected between August 2024 and March 2025.

The inclusion criteria for patients with stroke were: (1) meeting the diagnostic criteria for stroke (Chinese Society of Neurology & Chinese Stroke Society, 2019), (2) married, (3) stable vital signs and clear consciousness, and (4) capable of communicating through writing or speech. The inclusion criteria for spouse caregivers were: (1) being the patient’s spouse and primary caregiver (≥4 h/d), (2) stable vital signs and clear consciousness, and (3) capable of communicating through writing or speech. If either the patient or spouse did not agree to participate, the dyad was excluded from the study.

The exclusion criteria for patients with stroke and spouse caregivers were: (1) significant cognitive dysfunction (MMSE score < 21), (2) history of psychological disorders or psychiatric illness, and (3) presence of severe diseases such as heart, liver, or kidney failure, respiratory failure, or malignant tumors.

### Sample Size

According to the sample size requirements for network analysis, constructing the network model in this study required estimating 11 threshold parameters, with 55 pairwise correlation parameters (11 × [11−1]/2), totaling 66 parameters (Weiss et al., 2020). To ensure model stability, 3–5 participants per parameter are recommended (Weiss et al., 2020), resulting in a minimum sample size for this study of 198–330.

### Measurement

#### Demographic characteristics

General demographic characteristics for both dyad participants were obtained using a questionnaire and included age, educational level, working situation, and residence.

#### Self-Care of Chronic Illness Inventory

The Self-Care of Chronic Illness Inventory (SC-CII) was developed by Riegel et al. (2018), translated into Chinese by Yuan (2022), and validated by Jin et al. (2023). The SC-CII includes three subscales, namely self-care maintenance, self-care monitoring, and self-care management, and uses a 1–5 scoring system. Scores are summed and standardized separately for each subscale, with total subscale scores ranging from 0 to 100 and higher scores indicating better self-care capabilities (Riegel et al., 2018). In this study, Cronbach’s α values were .808 for self-care maintenance, .771 for self-care monitoring, and .807 for self-care management.

#### Caregiver Contribution to Self-Care Chronic Illness Inventory

The Caregiver Contribution to Self-Care Chronic Illness Inventory (CC-SC-CII) was developed by Vellone et al. (2020) and translated into Chinese by Chen et al. (2021). This inventory includes three subscales: caregiver contribution (CC) to self-care maintenance, CC to self-care monitoring, and CC to self-care management. Scoring and total score calculation are consistent that described for the SC-CII. In this study, Cronbach’s α values were .787 for CC to self-care maintenance, .765 for CC to monitoring, and .792 for CC to management.

#### Fear of Progression Questionnaire Short Form

The Fear of Progression Questionnaire Short Form (FoP-Q-SF), developed by Mehnert et al. (2006) and translated into Chinese by Wu (2017), includes 12 items across the two dimensions of physical health and social family. This scale uses a 1–5 scoring system, with a total possible scale score of 12–60, with scores ≥34 indicative of a significant level of fear that may suggest underlying psychological issues. The Cronbach’s α of the total scale and the dimensions of physical health and social family were, respectively, .849, .795, and .798 in this study.

#### Fear of Progression Questionnaire Short Form/Partner

The Fear of Progression Questionnaire Short Form/Partner (FoP-Q-P), developed by Zimmermann et al. (2011) and translated into Chinese by Wu (2017), includes 12 items in two dimensions: family health and social function. The scoring method and total score range are the same as described for FoP-Q-SF, with scores ≥34 indicative of a significant level of fear that may suggest underlying psychological issues. The Cronbach’s α of the total scale and the dimensions of family health and social function were, respectively, .870, .775, and .737 in this study.

### Ethical Statement

This study was approved by the Ethics Committee in Clinical Research of the First Affiliated Hospital of Wenzhou Medical University (No. KY2024-167) on July 26, 2024.

### Statistical Analysis

#### Preliminary analyses

First, descriptive analyses were conducted on demographic characteristics and each dimension, and sex-based differences in these variables were examined using the independent-sample *t* test, and the χ^2^ test. Because this study used self-reported information, Harman’s one-way factor model was used to verify the potential of common method variance. All of the above tests were conducted using SPSS 27.0 (IBM Corp., Armonk, NY, USA).

#### Network estimation and stability

*R* (version 4.4.3) was used to conduct the MNM. Before proceeding with further moderated network analysis, dimension scores were normalized using the *scale* function. MNM was constructed using the *modnets* package (version 0.9.0).

First, variable selection was performed using the *varSelect* function in the *modnets* package, and then MNM was estimated based on the variable selection objects using the *fitNetwork* function. Second, the function *plotNet* was used to visualize the network, and the AND rule was used, that is, connecting by solid edges only when both nodewise regressions between two dimensions pass the threshold and connecting by dashed edges only when both relevant interaction terms pass the significance threshold (Swanson, 2020). This means the presence of a solid line between two variables in a network visualization diagram indicates a bidirectional association between those variables. Third, the *condplot* function was used to plot the relationships between two variables at different levels of the moderator. Subsequently, expected influence (EI) was used to evaluate the importance of each dimension. We used the case-dropping bootstrap to directly quantify the stability of edge weights and centrality estimates. Correlation stability coefficients (CS-coefficients) were developed to quantify the stability of the indices, with CS-coefficients ≥.25 representing an acceptable degree of stability and those ≥.50 representing strong stability (Epskamp & Fried, 2018).

## Results

### Preliminary Analyses

The descriptive statistics results for the demographic characteristics and all dimensions for male and female patients as well as the full sample are given in Table 1. The descriptive statistics for spouses are given in Table 2. A common method bias test was conducted for self-assessment questionnaires. The results revealed 13 factors with characteristic roots >1, and the first factor explained 34.608%, which is below the critical value of 40%, indicating no serious common method bias.

**Table 1:** Descriptive Statistics for Patient Network Nodes

Variable	Male Patient (*n* = 282)	Female Patient (*n* = 102)	Total (*n* = 384)
	*n* (%)	*n* (%)	*n* (%)
Age (year)			
40–59	69 (24.5)	22 (21.6)	91 (23.7)
60–69	119 (42.2)	45 (44.1)	164 (42.7)
70–79	80 (28.4)	30 (29.4)	110 (28.6)
80–89	14 (5.0)	5 (4.9)	19 (4.9)
Educational level			
Primary school and below	179 (63.5)	75 (73.5)	254 (66.1)
Junior/High/vocational high school	88 (31.2)	21 (20.6)	109 (28.4)
College degree or above	15 (5.3)	6 (5.8)	21 (5.5)
Working situation			
Employed	73 (25.9)	22 (21.6)	95 (24.7)
Unemployed	27 (9.6)	5 (4.9)	32 (8.3)
Retired	182 (64.5)	75 (73.5)	257 (66.9)
Residence			
City	113 (40.1)	32 (31.4)	145 (37.8)
Countryside	169 (59.9)	70 (68.6)	239 (62.2)

	***M* (*SD*)**	***M* (*SD*)**	***M* (*SD*)**
Self-care of chronic illness			
Self-care maintenance	53.128 (15.614)	53.361 (16.175)	53.190 (15.744)
Self-care monitoring**	48.635 (18.479)	53.824 (16.458)	50.013 (18.089)
Self-care management*	57.240 (15.287)	52.614 (17.063)	56.011 (15.888)
Fear of disease progression			
Physical health	13.553 (4.016)	14.471 (4.622)	13.797 (4.199)
Social family	15.181 (3.429)	14.618 (4.505)	15.031 (3.747)

*Note*. The star symbol highlights significant differences between the sexes.

* *p* <.05

** *p* <.01.

**Table 2:** Descriptive Statistics for Spousal Network Nodes

Variable	Female Spouses (*n* = 282)	Male Spouses (*n* = 102)	Total (*n* = 384)
	*n* (%)	*n* (%)	*n* (%)
Age (year)			
40–59	88 (31.2)	21 (20.6)	109 (28.4)
60–69	109 (38.7)	38 (37.3)	147 (38.3)
70–79	70 (24.8)	36 (35.3)	106 (27.6)
80–89	15 (5.3)	7 (6.9)	22 (5.7)
Educational level			
Primary school and below	203 (72.0)	64 (62.7)	267 (69.5)
Junior/high/vocational high school	66 (23.4)	32 (31.4)	98 (25.5)
College degree or above	13 (4.6)	6 (5.9)	19 (4.9)
Working situation			
Employed	93 (33.0)	33 (32.4)	126 (32.8)
Unemployed	12 (4.3)	7 (6.9)	19 (4.9)
Retired	177 (62.8)	62 (60.8)	239 (62.2)
Residence			
City	113 (40.1)	32 (31.4)	145 (37.8)
Countryside	169 (59.9)	70 (68.6)	239 (62.2)

	***M* (*SD*)**	***M* (*SD*)**	***M* (*SD*)**
Caregiver contribution to self-care of chronic illness			
CC to self-care maintenance	55.99 (16.68)	54.31 (18.96)	55.54 (17.30)
CC to self-care monitoring*	56.72 (15.37)	53.04 (16.83)	55.74 (15.83)
CC to self-care management**	60.90 (13.52)	56.37 (14.41)	59.70 (13.89)
Fear of disease progression in partners			
Family health	16.05 (4.71)	16.11 (7.55)	16.07 (5.60)
Social function	8.01 (3.02)	8.36 (3.66)	8.10 (3.20)

*Note*. The star symbol highlights significant differences between the sexes. CC = caregiver contribution.

* *p* <.05.

** *p* <.01.

### Description of the Moderated Network Model

**Figure 2:**
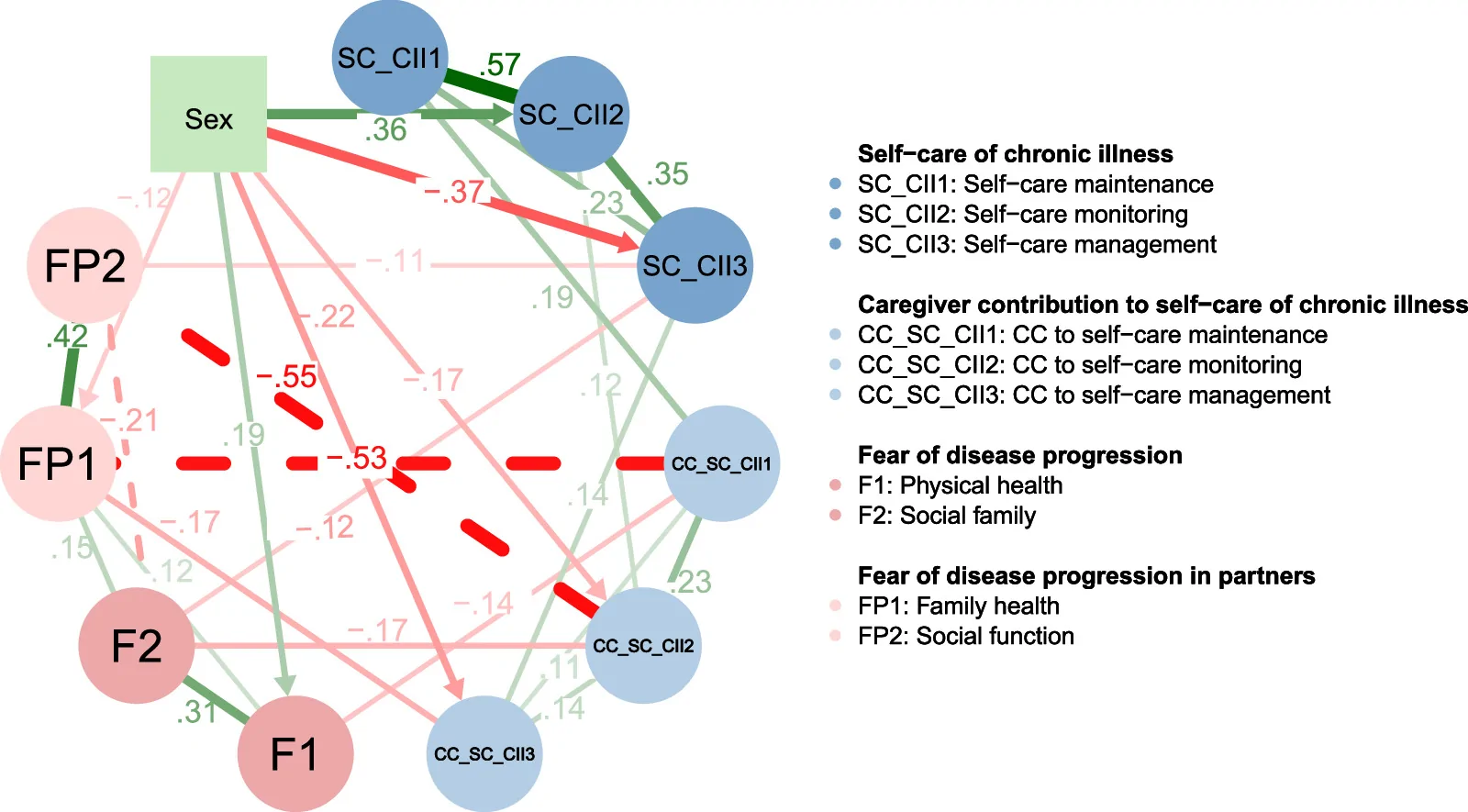
Moderated Network Analysis Visualization *Note*. A Green Solid Edge Indicates a Positive Correlation Between the Two Associated Nodes, and a Red Solid Edge Indicates a Negative Correlation. A Dashed Edge Indicates the Interactive Relation Between the Two Nodes Is Moderated by Sex. The Numerical Value Represents the Edge Weight of This Edge.

**Figure 3:**
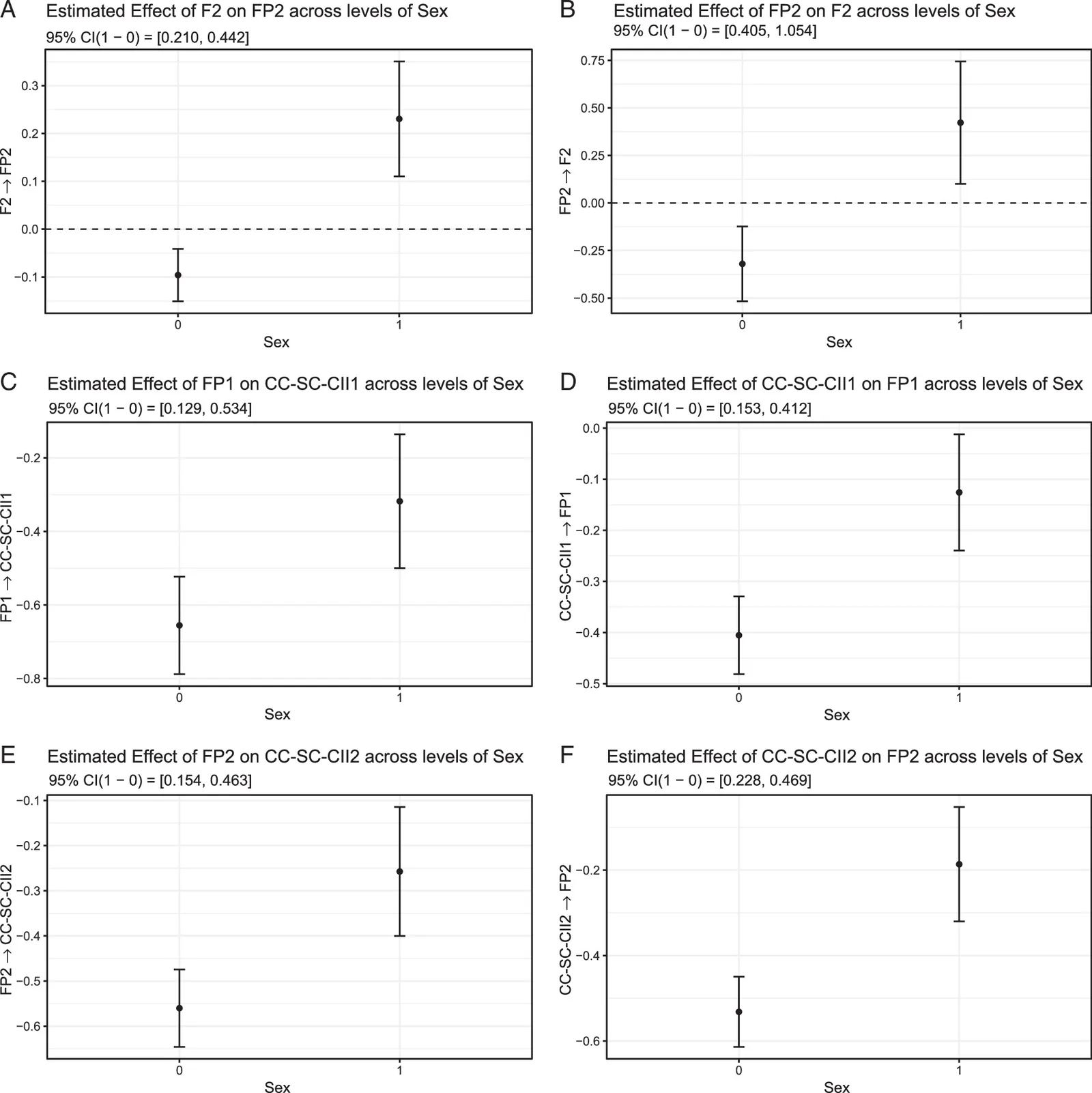
Plots of Conditional Effects *Note.* The 95% CI Displayed at the Top of the Graph Reflects the Coverage Interval of the Interaction Effect. If the 95% CI Does Not Include Zero, it Indicates the Moderating Effect Is Statistically Significant. 0 = Male Patients/Female Spouses, 1 = Female Patients/Male Spouses. For Example, in Figures (A and B), the Effect Is Negative for Male Patient–Female Spouse Dyads, But Positive in the Female Patient–Male Spouse Dyads. In Figures (C–F), the Negative Effects in the “0” Group Are Greater Than Those in the “1” Group. This Means That the Negative Effects on the Spouses of Male Patients (i.e., Female Spouses) Are Greater Than Those on the Spouses of Female Patients (i.e., Male Spouses). A, The Plot Shows the Conditional Effects of Patients’ Social Family × Sex on Spouses’ Social Function. Male Patients’ Social Family. B, The Plot Shows the Conditional Effects of Spouses’ Social Function × Sex on Patients’ Social Family. C, The Plot Shows the Conditional Effects of Spouses’ Family Health × Sex on Spouses’ Contribution to Self-Care Maintenance. D, The Plot Shows the Conditional Effects of Spouses’ Contribution to Self-Care Maintenance × Sex on Spouses’ Family Health. E, The Plot Shows the Conditional Effects of Spouses’ Social Function × Sex on Spouses’ Contribution to Self-Care Monitoring. F, The Plot Shows the Conditional Effects of Spouses’ Contribution to Self-Care Monitoring × Sex on Spouses’ Social Function.

As shown in Figure 2, the MNM revealed bidirectional relations between self-care in patients with stroke, spousal contributions to self-care, and FoP among couples as well as the moderating role of sex on these bidirectional relationships. The conditional effects are illustrated in Figure 3.

A correlation was identified between the dimensions of patient FoP (physical health, social family) and spouse FoP (family health, social function). Similarly, patient self-care (self-care maintenance, monitoring, management) was found to correlate with spousal contribution to self-care (spouses’ contribution to self-care maintenance, monitoring, and management). The specific findings are presented as follows: (1) patient FoP on physical health—spouse FoP on family health (*r* = .12; *r* in this study means edge weight, with larger values indicating a stronger connection between the two nodes), (2) patient FoP on social family—spouse FoP on family health (*r* = .15), (3) patient self-care maintenance—spouse contribution to self-care maintenance (*r* = .19), (4) patient self-care monitoring—spouse contribution to self-care monitoring (*r* = .12), and (5) patient self-care management—spouse contribution to self-care management (*r* = .14).

Furthermore, patient FoP on social family and spouse FoP on social function were found to be correlated, with this relationship moderated by sex. The 95% CI at the top of Figure 3A was [0.210, 0.442], excluding zero, indicating a statistically significant moderating effect. The 95% CI displayed at the top of the graph reflects the coverage interval of the interaction effect, and the moderating effect is considered statistically significant if the 95% CI does not include zero. The *x*-axis uses the following numerical coding: 0 = *male patient/female spouse group* and 1 = *female patient/male spouse group*. The estimated effects for group “0” in Figure 3A are negative, while those for group “1” are positive, indicating higher levels of FoP on social family in male patients are associated with lower levels of FoP on social function in their female spouses, while higher levels of FoP on social family in female patients are associated with lower levels of FoP on social function in their male spouses. Similarly, the 95% CI at the top of Figure 3B was [0.405, 1.054], excluding zero, indicating a statistically significant moderating effect. The estimated effects for group “0” in Figure 3B are negative, while those for group “1” are positive, indicating higher levels of FoP on social function in female spouses are associated with lower levels of FoP on social function in male patients, while higher levels of FoP on social function in male spouses are associated with lower levels of FoP on social function in female patients.

The dimensions of FoP in patients with stroke and their spouses were found to be correlated with the dimensions of dyadic self-care. The specific findings are presented as follows: (1) patient FoP on physical health—spouse contribution to self-care maintenance (*r* = -.14), (2) patient FoP on social family—patient self-care management (*r* = -.12), (3) patient FoP on social family—spouse contribution to self-care monitoring (*r* = -.17), (4) spouse FoP on family health—spouse contribution to self-care management (*r* = -.17), and (5) spouse FoP on social function—patient self-care management (*r* = −.11).

In addition, the association between spouse FoP and spouse contribution to self-care was identified as moderated by sex. The 95% CIs at the top of Figure 3C and D were [0.129, 0.534] and [0.153, 0.412], respectively, both excluding zero, indicating a statistically significant moderating effect. For both figures, the estimated effects for both group “0” and group “1” are negative, with the effect size in group “0” being more negative than that in group “1”, indicating that higher levels of FoP on family health in spouses are associated with lower patient contribution to self-care maintenance. Similarly, reduced patient contribution to self-care maintenance was found to be related to higher levels of FoP on family health in spouses. This negative effect was significantly stronger in the female spouse group than the male spouse group. Furthermore, the 95% CIs at the top of Figure 3E and F were [0.154, 0.463] and [0.228, 0.469]. Similarly, for both figures, the estimated effects for both group “0” and group “1” are negative, with the effect size in group “0” being more negative than that in group “1”, suggesting higher levels of FoP on social function in spouses to be associated with reduced patient contribution to self-care monitoring, while diminished patient contribution to self-care monitoring relates to increased FoP on social function in spouses. Notably, this reciprocal negative effect was significantly stronger in the female spouse group than the male spouse group.

### Centrality and Stability of the Moderated Network Model

Patient self-care maintenance exhibited the highest EI value (1.692). The CS coefficient for pairwise edge weights was .75, and the CS coefficient for interaction estimates was .28, showing the results of the edge weights to be stable. In addition, EI was stable, and the CS coefficients were .67/.36 (pairwise/interaction). All of the CS coefficients mentioned above were >.25, indicating acceptable network stability (Epskamp & Fried, 2018).

## Discussion

### Fear of Disease Progression and Dyadic Self-Care as Dyadic Phenomena

FoP on physical health and social family in patients with stroke was found to associate positively with their spouses’ FoP on family health, confirming FoP to be a dyadic phenomenon. Post-stroke, patients often worry about disease progression (Chun et al., 2022), leading to an FoP effect on physical health. Following stroke onset, patients often experience a loss of preexisting social roles as they transition into their new status as a care-recipient. Patients with stroke often fear becoming a burden to their family, leading to the observed FoP effect on social family. Emotional contagion theory suggests negative emotions from one partner’s adversity can spread within the marital relationship, increasing the perceived psychological distress of the other partner (Hatfield et al., 1993). Thus, patient FoP on physical health and social family can spread to the spouse, causing concern for the patient’s deteriorating health and leading to an FoP effect on family health. Similarly, when the spouse shows worry for the patient, the patient may sense this emotion, thereby exacerbating their own fear. Therefore, when developing intervention strategies, patients with stroke and their spouses should be regarded as an interconnected unit, with support provided to both to help them cope with and alleviate FoP jointly.

In addition, the finding that FoP on social family in male patients correlates negatively with FoP on social function in female spouses, while this correlation is positive for female patients and male spouses, is noteworthy. This may relate to sex-based differences in psychological coping styles and social roles. Female caregivers may suppress and conceal their negative emotions to avoid exacerbating the emotional burden of their care-recipient (Zhong et al., 2024). Thus, when male patients with stroke exhibit fear of social role loss and family burden due to illness, female spouses may suppress their fear of the patient’s social role impairment to avoid increasing the patient’s psychological burden. Moreover, in traditional Chinese societies, men are often expected to sustain their role as a “strong pillar” despite their illness (Liang & Li, 2024). Therefore, when female spouses show FoP, male patients may suppress their own emotions to avoid further triggering their spouse’s anxiety. In contrast, female patients tend to share emotions and concerns with their spouses, and couples may be more open to discussing disease fears and forming emotional resonance (Liang & Li, 2024). This emotional resonance strengthens the connection between the FoP of both spouses. In summary, when a patient is male, the couple’s emotional regulation is “complementary” and, when the patient is female, this regulation is “covariant.” In future interventions, for “male patient–female spouse” dyads, the couple should be guided to understand that suppressing emotions, while seemingly protective, can promote emotional distance. The goal should be to shift these couples from an “emotional complementarity” pattern to a “joint coping” mode. The intervener must proactively address the female spouse’s pressures and provide her with independent psychological support space while simultaneously helping the male patient redefine “strength,” emphasizing that honest communication and facing difficulties together are the true form of responsibility and encouraging them to actively inquire about their wife’s feelings. Conversely, for “female patient–male spouse” dyads, future interventions should focus on helping the couple transform shared emotional energy into positive, joint problem-solving actions while affirming their strength in open communication and remaining vigilant about the anxiety that excessive emotional resonance can bring. Emotion regulation skills should be taught so that, when one partner’s anxiety intensifies, the other can guide mindfulness breathing exercises to help calm emotions. Also, the male spouse should be trained to provide stable, rational emotional support and to avoid ineffective reassurance.

The maintenance, monitoring, and management of self-care in patients with stroke were shown to correlate positively with the contribution of their spouses to these respective self-care aspects, which is consistent with prior findings that dyadic self-care is a dyadic phenomenon (B. Liu et al., 2025; Lyons & Lee, 2018). Therefore, future interventions aimed at improving self-care in patients with stroke should target both the self-care capacity of the patient and the contributions of their caregiving spouse.

### Bidirectional Relationship Between Fear of Disease Progression and Dyadic Self-Care

Patient FoP on physical health was found to be bidirectionally and negatively associated with spouse contribution to self-care maintenance, and patient fear of “social family” was also found to be bidirectionally and negatively associated with spouse contribution to self-care monitoring. The findings of previous studies indicate family caregivers often experience heavy caregiving burdens and are prone to compassion fatigue due to continuous and high-intensity caregiving coupled with long-term exposure to an environment of exhaustion and trauma, which may lead to their avoidance of caregiving responsibilities (Liao et al., 2022). Thus, when patients with stroke repeatedly express intense fear of symptom deterioration, their spouses may be chronically exposed to these negative emotions and caregiving stress. This may then lead to caregiver burnout and compassion fatigue, resulting in reduced suggestions and monitoring of healthy behaviors and a decrease in their contributions to self-care maintenance and monitoring, which may result in the patient failing to receive adequate daily care support. Furthermore, with less monitoring from the spouse, the patient may lack the necessary supervision and guidance in self-care, making it easier to overlook changes in their condition. These factors ultimately increase patient fear of further deterioration in physical health and social family.

Patient FoP on social family was shown to correlate bidirectionally and negatively with their self-care management. The findings of previous studies indicate anxiety in patients with anxiety disorders correlates negatively with their self-care (Cao et al., 2023). Similarly, patients with stroke may neglect their own care needs due to concerns about burdening their family or lack the motivation to engage in effective self-care due to worries about their future role. When patients are unable to manage their condition and daily life effectively, they may feel incapable of fulfilling their family responsibilities, thereby exacerbating their FoP on social family.

In addition, spouse FoP on social function was shown to correlate bidirectionally and negatively with the patient's self-care management. The findings of previous studies indicate that being in a close spousal relationship leads caregivers to view protecting their partners from harm as their duty, which can result in overprotection (M. D. Johnson et al., 2015). In adult patients, overprotection can exacerbate their sense of helplessness and diminish autonomy (Zniva et al., 2017). When spouses exhibit excessive fear of social function, they may engage in overprotection, which can make the patient feel their capabilities are being undermined, leading to a reluctance to actively participate in self-care management measures. Conversely, when the patient’s self-care management ability is insufficient, the spouse may worry about further disease progression that would make the patient unable to assume family and social responsibilities, resulting in fear of social function.

Also, spouse FoP on family health was found to correlate bidirectionally and negatively with their contribution to self-care management. The higher a caregiver’s level of anxiety, the lower their involvement in the patient’s self-care management and the lower their ability to accurately assess the patient’s condition and recognize symptoms (Buck et al., 2015). Thus, when spouses experience fear and anxiety about deteriorating patient health, the psychological burden related to this consumes a significant amount of their energy and emotional resources. As a result, they may struggle to focus on identifying changes in patient symptoms and fail to respond in a timely and effective manner, thus impairing their performance in self-care management. When the spouse’s contribution to self-care management is insufficient, the patient may feel unsupported and unloved, which can negatively impact the couple’s relationship and family harmony, potentially further exacerbating spousal worries about the patient’s health and the family’s future even more, stoking fear for “family health.”

In summary, FoP and dyadic self-care in patients with stroke and their spouses were found to be bidirectionally and negatively associated. This finding suggests patients and their spouse caregivers should be regarded as one dynamic, integrated system and addressed using interventions that integrate both skill-based and positive psychological components (Bakas et al., 2022). Importantly, spousal overprotection should be avoided, and patients should be encouraged within the limits of their capabilities to participate in household chores or social activities. Moreover, the patient self-care maintenance dimension was identified as the most impactful on the relationship between FoP and dyadic self-care, suggesting interventions should place particular emphasis on this aspect through, for example, promoting medication adherence and consistent rehabilitation exercises.

### Sex as a Moderator of the Relationship Between Fear of Disease Progression and Dyadic Self-Care

The negative impact of female spouse FoP on their contribution to self-care is significantly stronger than that of male spouse FoP. Similarly, the negative effect of contribution to self-care on FoP is also significantly stronger in female spouses than male spouses. Within Chinese families, men typically shoulder heavier economic support, decision-making, and protective burdens (Qing, 2020). A sudden stroke may prevent them from continuing to fulfill their expected roles, causing distress not only for patients but for their female spouses as well, who may worry about the future of the family. Influenced by traditional Chinese culture, the phenomenon of “feminization of caregiving responsibilities” is quite pronounced (Liang & Li, 2024). Female caregivers accept higher caregiving tasks and expectations passively, potentially leading to psychological pressures and more negative emotions. Moreover, female spouses are not only expected to perform the actual caregiving tasks but also to provide emotional support, undertaking more “emotional labor” as a result (Liang & Li, 2024). This unquantified and invisible emotional labor, while demanding a significant expenditure of psychological energy from women, often goes unrecognized and undervalued. Caregiving stresses, negative emotions, and invisible emotional labor constantly trouble female spouses, leading them to focus more on their emotional responses and coping strategies. As a result, they may devote less energy and time to encouraging patients with stroke to maintain self-care behaviors and to monitoring their self-care efficacy. When female spouses perceive they are not contributing sufficiently to the self-care maintenance of their patient, they may feel guilt toward the patient and the family, thereby increasing their concerns for “family health”. Similarly, when their monitoring of the patient’s symptoms and signs decreases (i.e., reduced contribution to self-care monitoring), they may fail to promptly detect changes in patient condition and thus not implement effective and timely intervention measures. This may lead to greater worry and fear about the patient’s future and elevate fear of “social function.”

In summary, male and female spouses may both fall into a vicious cycle of “increased fear—reduced self-care contributions,” with female spouses being more significantly affected by this cycle. Therefore, interventions should be developed that guide female spouses to identify and manage invisible emotional labor (e.g., forced cheerfulness and suppressed worries), learn to establish “emotional breaks,” and share emotional support responsibilities with other family members or support systems. These interventions should also help female spouse caregivers to recognize that reduced caregiving due to limited energy is not their personal failure but rather the result of an inadequate family support system. In addition, support groups for female spouses should be established to provide spaces for emotional expression and experience sharing. Furthermore, remote monitoring technology (e.g., simple devices for medication reminders and blood pressure tracking) may be introduced to alleviate the continuous monitoring burden faced by female spouses while maintaining the ability to regularly monitor the patient’s condition and reducing the fears stoked by lack of sufficient information.

### Limitations

This study was affected by several limitations. First, as all participants were recruited from a single hospital, the findings may not be generalizable to other health care institutions and settings. Thus, multicenter studies are needed. Second, the cross-sectional design employed precludes causal inferences about FoP and dyadic self-care in patients with stroke and their spouses. Future longitudinal studies should be conducted to elucidate these causal relationships more effectively.

### Conclusions

The results of this study support that fear of disease progression in both patients with stroke and their spouse caregivers is related dyadically to dyadic self-care and that fear of disease progression is bidirectionally and negatively associated with dyadic self-care and moderated by sex. When patients are male, emotional regulation within the patient-spouse caregiver relationship reflects a “complementary” dynamic. When patients are female, emotional regulation within this relationship reflects a “covariant” dynamic. Finally, excessive fear in female spouses more strongly and negatively impacts their contribution to self-care than excessive fear in male spouses.
